# P-1484. *In vitro* Activity of Cefiderocol and Comparator Agents Against Non-fermentative Gram-negative Bacilli Isolated from Patients Hospitalized with Pneumonia as part of the SENTRY Global Surveillance Program (2020-2022)

**DOI:** 10.1093/ofid/ofae631.1654

**Published:** 2025-01-29

**Authors:** Frank Kung, Sean T Nguyen, Boudewijn L DeJonge, Christopher M Longshaw, Jason J Bryowsky, Joshua Maher, Rodrigo E Mendes, Miki Takemura, Yoshinori Yamano

**Affiliations:** Shionogi Inc, Basking Ridge, New Jersey; Shionogi Inc., Florham Park, New Jersey; Shionogi Inc., Florham Park, New Jersey; Shionogi B.V., London, England, United Kingdom; Shionogi Inc., Florham Park, New Jersey; Element Materials Technology/Jones Microbiology Institute, North Liberty, Iowa; JMI Laboratories, North Liberty, Iowa; Shionogi & Co., Ltd, Toyonaka, Osaka, Japan; Shionogi & Co., Ltd., Toyonaka, Osaka, Japan

## Abstract

**Background:**

Cefiderocol (CFDC) is a siderophore-conjugated cephalosporin with broad activity against Gram-negative bacteria including non-fermenters resistant to carbapenems. The *in vitro* activity of CFDC and comparators was evaluated against glucose non-fermenting Gram-negative bacteria isolated from patients hospitalized with pneumonia.

**Table 1:**
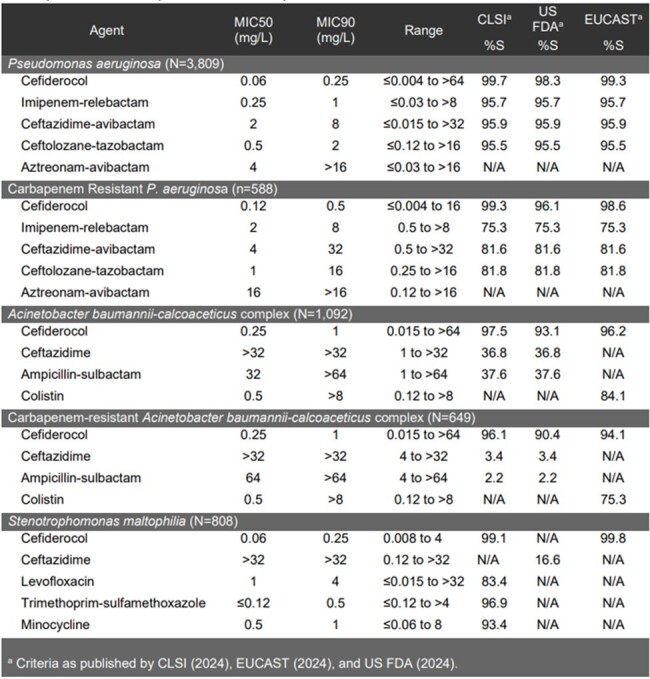
Activity of cefiderocol and comparator agents against Pseudomonas aeruginosa, Acinetobacter baumannii-calcoaceticus complex, and Stenotrophomonas maltophilia isolates from patients hospitalized with pneumonia from 2020-2022

**Methods:**

5,995 isolates were collected between 2020-2022 from pneumonia patients as part of the global SENTRY Antimicrobial Surveillance Program, including: 3,809 *Pseudomonas aeruginosa* (PSA), 1,092 *Acinetobacter baumannii-calcoaceticus* complex (ABC), 808 *Stenotrophomonas maltophilia* (STM), 104 *Achromobacter spp.* (ACHR), 81 *Burkholderia cepacia* complex (BURK), and 101 other isolates. Minimum inhibitory concentrations (MIC) were assessed by CLSI guidelines using broth microdilution with cation-adjusted Mueller-Hinton broth (CAMHB) for comparators and iron-depleted CAMHB for CFDC. Susceptibility was assessed according to 2024 CLSI, FDA, and EUCAST breakpoints. Carbapenem resistance (CR) was defined as resistant to meropenem and imipenem by CLSI breakpoints.

**Results:**

Against PSA, CFDC and β-lactam/β-lactamase inhibitor combinations all had susceptibility >95%. However only CFDC maintained high susceptibility against CR-PSA with 99.3% of the isolates being susceptible by CLSI breakpoints. No other comparator had > 90% susceptibility against CR-PSA (Table 1). Against ABC, CFDC demonstrated 97.5% susceptibility by CLSI breakpoints. This high susceptibility was maintained against CR-ABC with 96.1% susceptibility using CLSI breakpoints. Colistin showed 75.5% susceptibility per EUCAST breakpoint, but ceftazidime and ampicillin-sulbactam had no appreciable activity. CFDC, trimethoprim-sulfamethoxazole, and minocycline all showed >90% susceptibility against STM. Against ACHR and BURK, CFDC had high activity, with MIC_50/90_ values of 0.03/0.5 and 0.06/1 mg/L, respectively.

**Conclusion:**

CFDC demonstrated high *in vitro* activity against glucose non-fermenters isolated from hospitalized patients with pneumonia. In particular, CFDC maintained high susceptibility against CR isolates making it an important treatment option for pneumonia patients at risk of infection with a CR non-fermenter.

**Disclosures:**

**Frank Kung, PhD**, Shionogi Inc: Employee **Sean T. Nguyen, PharmD**, Shionogi Inc.: Employee **Boudewijn L. DeJonge, PhD**, Shionogi Inc.: Employee **Christopher M. Longshaw, PhD**, Shionogi BV: Employee **Jason J. Bryowsky, PharmD, MS**, Shionogi: Employee **Rodrigo E. Mendes, PhD**, GSK: Grant/Research Support **Miki Takemura, n/a**, Shionogi & Co., Ltd.: Employee **Yoshinori Yamano, PhD**, Shionogi & Co., Ltd.: Employee

